# Status of vitamin D, antimicrobial peptide cathelicidin and T helper-associated cytokines in patients with diabetes mellitus and pulmonary tuberculosis

**DOI:** 10.3892/etm.2014.2042

**Published:** 2014-10-31

**Authors:** YUNFEI ZHAN, LING JIANG

**Affiliations:** 1 Department of Endocrinology, Qilu Hospital, Shandong University, Jinan, Shandong 250012, P.R. China; 2 Department of Internal Medicine, Shandong Provincial Chest Hospital, Jinan, Shandong 250013, P.R. China

**Keywords:** vitamin D, antimicrobial peptide cathelicidin, T helper 17, cytokine, diabetes mellitus, pulmonary tuberculosis

## Abstract

Pulmonary tuberculosis (PTB) is a high burden infectious disease in China. The immune function is damaged in patients with diabetes mellitus (DM) who are easy to infect with *Mycobacterium tuberculosis* (Mtb). The growth of Mtb has been shown to be restrained following the administration of vitamin D and antimicrobial peptide cathelicidin (LL-37); however, the effect in patients with DM and PTB remains unclear. Vitamin D can regulate the immune system through Vitamin D receptors expressed in T helper (Th) cells. The aim of the present study was to analyze the status and correlations of vitamin D, LL-37 and Th-associated cytokines in patients with PTB or PTB with DM (DMPTB). Serum 25-hydroxyvitamin D_3_ [25(OH)D_3_] levels were measured by liquid chromatography-tandem mass spectrometry, while plasma LL-37 levels were analyzed using a solid-phase enzyme-linked immunosorbent assay. Flow cytometry was used to analyze the levels of Th cytokines, including Th1-associated IFN-γ, Th2-associated IL-4 and Th17-associated IL-17. The results revealed that patients with PTB and DMPTB were vitamin D deficient or had insufficient vitamin D levels. Furthermore, the levels of LL-37, IFN-γ, IL-4 and IL-17 were higher in the PTB and DMPTB groups when compared with the normal controls. These results indicated that vitamin D supplementation is necessary for PTB and DMPTB patients. In addition, LL-37, IFN-γ and IL-17 may be diagnostic indexes that become elevated in the compensatory response caused by Mtb infection. Vitamin D can regulate the immune status in patients suffering from PTB.

## Introduction

*Mycobacterium tuberculosis* (Mtb) is a major international public health problem, with one-third of the world’s population latently infected by Mtb. In these cases, active tuberculosis (TB) disease will develop following failure of their immune system (1,2). The age-standardized prevalence rates of total diabetes and prediabetes were 9.7% (males, 10.6%; females, 8.8%) and 15.5% (males, 16.1%; females, 14.9%), respectively, in China (3). Immune function may decrease in diabetes mellitus (DM) subjects who are susceptible to pulmonary tuberculosis (PTB). Therefore, increased research should focus on cases of DM with PTB (DMPTB).

Serum 25-hydroxyvitamin D_3_ [25(OH)D_3_] is a valid measure of the vitamin D status (4). 1,25(OH)_2_D_3_ is an active form of vitamin D_3_ that functions as an immunomodulator in immune homeostasis (5,6), and has been shown to restrain the growth of Mtb (7). Deficient serum vitamin D levels have been associated with the incidence of TB (6,8,9) and DM (10,11), while sufficient serum 25(OH)D_3_ levels have been shown to protect against TB or DM (5,12). The interaction of 1,25(OH)_2_D_3_ with its receptor on T lymphocytes may play an important role in the regulation of the immune status (13). A number of studies have indicated that 1,25(OH)_2_D_3_ may be a successful anti-TB therapeutic agent (7,14).

Antimicrobial peptide cathelicidin (LL-37), an endogenous antimicrobial peptide synthesized by neutrophils, monocytes, T cells and other immune cells in a vitamin D-dependent manner, has been demonstrated to function against Mtb infection using *in vitro* and *in vivo* models (15–17). In addition, vitamin D can restrict the acquired immune response against TB by regulating the production of cytokines. 1,25(OH)_2_D_3_ has been shown to differentially modulate the production of cytokines in response to Mtb antigens by predominantly suppressing interferon (IFN)-γ production in a dose-dependent manner (18). Furthermore, a number of studies (19,20) have indicated that interleukin (IL)-17A plays a critical role in the prevention of Mtb infection via the induction of mature granuloma formation.

However, the status of 25(OH)D_3_ and LL-37 is not clear in patients with PTB complicated with DM. To the best of our knowledge, T helper (Th)-associated cytokines, including IFN-γ, IL-4 and IL-17, have not been analyzed in patients with DMPTB. Therefore, the aim of the present study was to investigate the status of 25(OH)D_3_, LL-37 and Th-associated cytokines, in order to evaluate the association with PTB or DMPTB.

## Materials and methods

### Study population and specimen collection

In total, 90 participants were enrolled in the study. The study protocol was approved by the Clinical Research Ethics Committee of Shandong Provincial Chest Hospital (Jinan, China), and informed consent was obtained from every participant who provided blood samples voluntarily for the study. DM was diagnosed by the following parameters: Fasting plasma glucose (FPG) level of ≥126 mg/dl (7.0 mmol/l), symptoms of hyperglycemia, casual plasma glucose level of ≥200 mg/dl (11.1 mmol/l) and 2 h plasma glucose level of ≥200 mg/dl (11.1 mmol/l), measured during an oral glucose tolerance test. PTB was diagnosed by a positive sputum acid-fast bacillus smear or a typical PTB image from a chest computed tomography scan, and an effective outcome following anti-TB therapy. Individuals were excluded from the study if they exhibited a clinical manifestation of infection or had been administered corticosteroids, diuretics or supplementary vitamin D_3_ in the three months previously. The patients were divided into three groups, which included 30 normal controls (NC group), 30 PTB patients (PTB group) and 30 DM and PTB patients (DMPTB group). The NC group comprised volunteers, while the PTB and DMPTB groups consisted of inpatients from the Shandong Provincial Chest Hospital. Patient characteristics, including age, gender and FPG, were collected. The blood sample from patients fasted for 10 h was collected using EDTA K_2_ tubes and separated in gel coagulating tubes (Shandong Chengwu Yongkang Medical Products Company, Chengwu, China). All fresh specimens were transported on ice, and the plasma and serum samples were separated within 20 min by centrifugation at 1,500 × g at 4°C for 15 min. The EDTA plasma samples required for LL-37 analysis and the serum samples used for vitamin D_3_ analysis were stored in fresh polypropylene tubes at −70°C. The serum samples required for cytokines analysis were stored at −20°C.

### 25(OH)D_3_ measurement

Serum samples were stored at −70°C. Reagents, including methanol, methyl cyanides, n-hexane and anhydrous ethanol (Merck KGaA, Darmstadt, Germany), standard substance (SRM972, Sigma-Aldrich, St. Louis, MO, USA), internal standard product (Advanced Medical Isotopes Corporation, Kennewick, WA, USA) and quality control (RECIPE, Munich, Germany). Serum 25(OH)D_3_ levels were measured by liquid chromatography-tandem mass spectrometry on an API 4000 mass spectrometer (Applied Biosystems Life Technologies, Foster City, CA, USA) in the Jinyu Medical Test Center (Guangzhou, China).

### Human LL-37 measurement

A solid-phase enzyme-linked immunosorbent assay (HK321, HyCult Biotechnology, Uden, Netherlands) was used to measure the levels of LL-37. According to the manufacturer’s instructions, the samples and standards were incubated in microtiter wells coated with mouse monoclonal antibodies recognizing against human LL-37 that were provided with the kit. A biotinylated tracer antibody that was conjugated to streptovidin-peroxidase was used to bind to the human LL-37. The conjugated streptovidin-peroxidase reacted with the substrate and tetramethylbenzidine, and the enzyme reaction was stopped following the addition of oxalic acid. Absorbance was measured at 450 nm with a spectrophotometer. A standard curve was obtained by plotting the absorbance values against the corresponding concentrations of the human LL-37 standards. The concentration of human LL-37 in the samples, which were run concurrently with the standards, was determined from the standard curve.

### Cytokine measurement

A human Th1/Th2/Th9/Th17/Th22 13 Plex FlowCytomix kit (BMS817FF, eBioscience, Inc., San Diego, CA, USA) was used to determine the levels of the various cytokines by flow cytometry (FACSCalibur; BD Biosciences, Franklin Lakes, NJ, USA). Analysis was performed with FlowCytomix^TM^ Pro 3.0 software (eBioscience, Inc.). The principle of this test was the fluorescent bead immunoassay. Fluorescent beads were conjugated to each target analyte followed by addition of a biotin-conjugated secondary detection antibody and a dye that functions as a reporter. In this experiment streptavidin-conjugated phycoerythrin dye was used and the different intensities of the fluorescent beads bound to the antibody were measured. The concentration of the various analytes was calculated using a standard curve.

### Statistical analysis

Results are expressed as the mean ± standard deviation and statistical analyses were performed using SPSS 14.0 for Windows (SPSS, Inc. Chicago, IL, USA). Comparisons between mean values were analyzed by one-way analysis of variance using the least significant difference test and Tamhane’s T2 test, while categorical variables were analyzed using the χ^2^ test. Correlation analyses were assessed using Pearson’s test. P<0.05 was considered to indicate a statistically significant difference.

## Results

### Participants and clinical parameters

A total of 90 participants were enrolled in the study, which comprised three groups (NC, PTB and DMPTB). In the NC group, the mean age was 38.83±12.88 years, and the male/female ratio was 14/16. In the PTB group, the mean age was 36.83±16.02 years, and there were 18 males and 12 females. In the DMPTB group, the mean age was 43.90±12.85 years, and the male/female ratio was 14/16. No statistically significant differences were observed among the groups with regard to the mean age or male/female ratio (P>0.05). However, the FPG level in the DMPTB group (11.05±3.62 mmol/l) was significantly higher compared with the NC (4.73±0.55 mmol/l, P<0.001) and PTB groups (4.80±0.54 mmol/l, P<0.001). A statistically significant difference was not observed in the FPG between the NC and PTB groups (P=0.952). The data are summarized in Table I.

### Concentration of serum 25(OH)D_3_ and plasma LL-37

**Figure 1:**
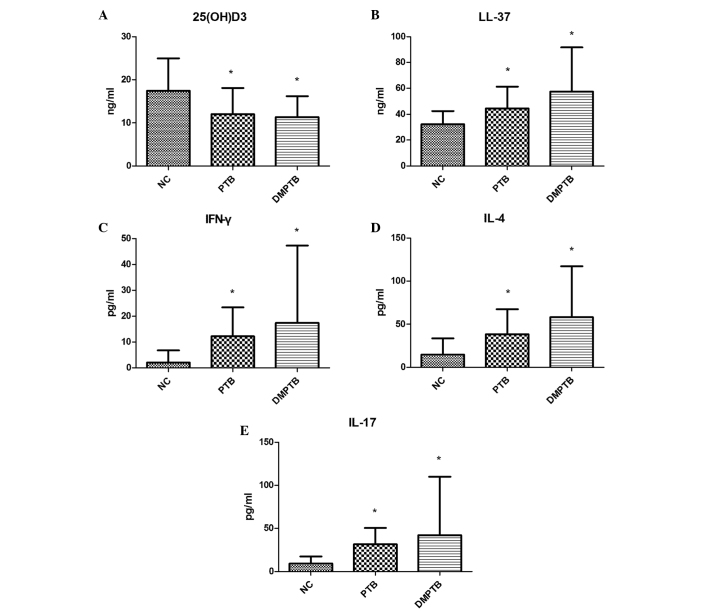
Concentration of (A) serum 25(OH)D_3_, (B) plasma LL-37, serum (C) IFN-γ, (D) IL-4 and (E) IL-17 in the NC, PTB and DMPTB groups. Serum levels of 25(OH)D_3_ significantly decreased, while plasma levels of LL-37 and serum levels of IFN-γ, IL-4 and IL-17 markedly increased in the PTB and DMPTB groups when compared with the NC group. ^*^P<0.05, vs. NC. NC, normal control; PTB, pulmonary tuberculosis; DMPTB, diabetes mellitus and pulmonary tuberculosis; 25(OH)D_3_, 25-hydroxyvitamin D_3_; IL, interleukin; IFN, interferon; LL-37, antimicrobial peptide cathelicidin.

Serum 25(OH)D_3_ concentrations in the NC group (17.49±7.50 ng/ml) were markedly higher compared with the levels in the PTB (12.04±6.08 ng/ml; P<0.01) and DMPTB groups (11.36±4.85 ng/ml; P<0.01). No statistically significant difference was observed in the 25(OH)D_3_ concentration between the PTB and DMPTB groups. In addition, the plasma level of LL-37 in the NC group (32.20±10.14 ng/ml) was significantly lower compared with the PTB (44.53±16.88 ng/ml) and DMPTB groups (57.52±34.17 ng/ml; P<0.01). The concentration of plasma LL-37 was not significantly different between the DMPTB and PTB groups (P=0.950; Table II; Fig. 1A and B).

### Levels of Th-associated cytokines

Serum levels of IFN-γ, IL-4 and IL-17 in the PTB group (12.23±11.18, 38.37±28.98 and 31.56±19.07 pg/ml, respectively) and DMPTB group (17.43±29.90, 58.18±58.96 and 42.24±67.70 pg/ml, respectively) were found to be significantly higher compared with the levels in the NC group (2.09±4.66, 14.72±18.86 and 9.33±8.15 pg/ml, respectively; P<0.05). In addition, no statistically significant difference was observed between the PTB and DMPTB groups (P>0.05; Table II; Fig. 1C–E).

### Correlations among vitamin D, LL-37 and Th-associated cytokines in the PTB and DMPTB patients

Correlations among the levels of vitamin D, LL-37 and Th-associated cytokines are summarized in Table III. The IFN-γ level was found to be negatively correlated with the LL-37 concentration (r=−0.379, P<0.001), but strongly positively correlated with IL-4 (r=0.616, P<0.001) and IL-17 (r=0.790, P<0.001). In addition, a significant positive correlation was observed between the levels of IL-4 and IL-17 (r=0.580, P<0.001). However, no significant correlation was identified between the vitamin D concentration and the levels of LL-37 (r=0.226, P=0.082), IFN-γ (r=−0.075, P=0.568), IL-4 (r=0.030, P=0.818) and IL-17 (r=0.064, P=0.627). Furthermore, no statistically significant correlation was identified between the level of LL-37 and IL-4 (r=−0.182, P=0.164) or IL-17 (r=−0.052, P=0.694).

## Discussion

The host immune system plays an important role in the development of PTB. Novel immune strategies are crucial to the diagnosis and therapy of PTB, and the correlation between vitamin D levels and the immune status has resulted in an increasing number of studies investigating vitamin D.

Vitamin D is a type of hormone that functions in the maintenance of immune homeostasis (6,8). A limited number of studies have been published investigating the levels of vitamin D and LL-37 in patients with DM and PTB. Thus, the present study analyzed the levels of vitamin D and LL-37 in NC, PTB and DMPTB patients. The concentration of vitamin D in the NC group was significantly higher compared with the PTB and DMPTB groups. A previous study (21) reported that the mean 25(OH)D_3_ levels for the entire population were in the ‘insufficient’ range (21.3±9.78 ng/ml), but were observed to be lower in PTB patients (21). Vitamin D deficiencies were observed in the PTB and DMPTB patients in the present study. Previous studies (4,15) have demonstrated that individuals who are vitamin D deficient or have insufficient levels of vitamin D are more susceptible to Mtb infections. Hypovitaminosis D may predispose individuals to multidrug-resistant TB (MDR-TB) and increase the time in which MDR-TB sputum smear negativity is achieved (22).

In the present study, the plasma level of LL-37 was 44.53±16.88 ng/ml in the PTB group. Yamshchikov *et al* found that the mean serum LL-37 concentration for PTB patients was 49.5 ng/ml, and that the serum vitamin D level exhibited no correlation with the plasma LL-37 level (15). Stored serum specimens used in the study by Yamshchikov differed from the EDTA plasma samples of fresh blood transported on ice used in the present study. Temperature can influence the results of the blood LL-37. However, the results of the LL-37 blood levels were similar in the two studies. In the current study, the serum 25(OH)D_3_ and plasma LL-37 levels were not shown to correlate.

Plasma levels of LL-37 in the PTB (44.53±16.88 ng/ml) and DMPTB groups (57.52±34.17 ng/ml) were significantly higher compared with the NC group in the present study (P<0.01). Yamshchikov *et al* hypothesized that higher LL-37 concentrations correlated with acid fast bacilli sputum smear positivity. Thus, the circulating LL-37 level may be a potential biomarker in patients with active TB disease (15). The results of the present study also support the hypothesis that elevated plasma levels of LL-37 in PTB patients may be a potential biomarker for PTB. Furthermore, an additional study indicated that administration of oral 4-phenylbutyrate with vitamin D_3_ induces LL-37 peptide expression in functional immune cells and enhances intracellular Mtb death in macrophages (23).

In the present study, IFN-γ, IL-4 and IL-17 levels in the PTB and DMPTB patients were found to markedly increase when compared with the NC group. IFN-γ exhibited a positive correlation with IL-17, and was shown to negatively correlate with LL-37. Previous studies have demonstrated that vitamin D may downregulate the recruitment and activation of T cells at infection sites, including those of PTB (24,25). Furthermore, a previous study reported that patients with DMPTB were characterized by elevated frequencies of Th1 and Th17 cells, which may contribute to the increased immune pathology observed in Mtb infections (26). The present study considered IFN-γ and IL-17 as a compensatory response that enhanced the anti-inflammatory reaction and as an excessive immune reaction that accelerated the damage in the PTB and DMPTB patients. An associated study indicated that the specific secretion of soluble immunological factors, in addition to IFN-γ, may be used to evaluate Mtb infection and TB (27). Thus, IFN-γ is a good marker for Mtb infection (28).

Vitamin D supplements for chronic inflammation have been a prospective research subject in recent years. A number of studies have demonstrated that vitamin D supplements are pertinent in the treatment of TB (5,21). In addition, vitamin D has been demonstrated to significantly hasten sputum culture conversion in participants with the TT genotype of the TaqI vitamin D receptor polymorphism (29). Therefore, future studies investigating vitamin D therapy for DM with PTB are required. The use of LL-37 and IL-17 as new biomarkers need more specimens in order to verify their feasibility for future studies.

In conclusion, lower serum levels of vitamin D were observed in patients with PTB, particularly those also suffering from DM. In addition, the plasma levels of LL-37, serum IFN-γ and IL-17 increased in the compensatory response observed in PTB patients. Therefore, LL-37 and IL-17 may serve as potential biomarkers for the diagnosis of TB, and vitamin D may be used as a potential adjuvant treatment for TB.

## Figures and Tables

**Table I:** Clinical characteristics of the participants.

Group	Cases, n	Age, years (mean ± SD)	Gender, n (male/female)	FPG, mmol/l
NC	30	38.83±12.88	14/16	4.73±0.55
PTB	30	36.83±16.02	18/12	4.80±0.54
DMPTB	30	43.90±12.85	14/16	11.05±3.62^a^

FPG, fasting plasma glucose; NC, normal control; PTB, pulmonary tuberculosis; DMPTB, diabetes mellitus and pulmonary tuberculosis; SD, standard deviation.

a P<0.001 vs. NC group and PTB group.

**Table II:** Levels of 25(OH)D_3_, LL-37 and cytokines in the blood.

Index	NC (n=30)	PTB (n=30)	DMPTB (n=30)
25(OH)D_3_, ng/ml	17.49±7.50	12.04±6.08^a^	11.36±4.85^a^
LL-37, ng/ml	32.20±10.14	44.53±16.88^a^	57.52±34.17^a^
IFN-γ, pg/ml	2.09±4.66	12.23±11.18^b^	17.43±29.90^b^
IL-4, pg/ml	14.72±18.86	38.37±28.98^b^	58.18±58.96^b^
IL-17, pg/ml	9.33±8.15	31.56±19.07^b^	42.24±67.70^b^

25(OH)D_3_, 25-hydroxyvitamin D_3_; LL-37, antimicrobial peptide cathelicidin; IFN, interferon; IL, interleukin; NC, normal control; PTB, pulmonary tuberculosis; DMPTB, diabetes mellitus and pulmonary tuberculosis.

a P<0.01 vs. NC group;

b P<0.05 vs. NC group.

**Table III:** Correlations among vitamin D, LL-37 and Th-associated cytokines in patients with PTB and DMPTB.

Index	LL-37	IL-4	IL-17	IFN-γ
IFN-γ	r=−0.379	r=0.616	r=0.790	-
	P<0.001	P<0.001	P<0.001	-
IL-4	r=−0.182	-	r=0.580	r=0.616
	P=0.164	-	P<0.001	P<0.001
Vitamin D	r=0.226	r=0.030	r=0.064	r=−0.075
	P=0.082	P=0.818	P=0.627	P=0.568
IL-17	r=−0.052	r=0.580	-	r=0.790
	P=0.694	P<0.001	-	P<0.001

LL-37, antimicrobial peptide cathelicidin; IFN, interferon; IL, interleukin; Th, T helper; PTB, pulmonary tuberculosis; DMPTB, diabetes mellitus and pulmonary tuberculosis.
